# Complement and endothelial cell activation in COVID-19 patients compared to controls with suspected SARS-CoV-2 infection: A prospective cohort study

**DOI:** 10.3389/fimmu.2022.941742

**Published:** 2022-09-20

**Authors:** Flavio Bruni, Panteleimon Charitos, Maurin Lampart, Stephan Moser, Martin Siegemund, Roland Bingisser, Stefan Osswald, Stefano Bassetti, Raphael Twerenbold, Marten Trendelenburg, Katharina M. Rentsch, Michael Osthoff

**Affiliations:** ^1^ Division of Internal Medicine, Department of Biomedicine and Department of Clinical Research, University Hospital Basel, University of Basel, Basel, Switzerland; ^2^ Department of Cardiology and Cardiovascular Research Institute Basel (CRIB), University Hospital Basel, University of Basel, Basel, Switzerland; ^3^ Intensive Care Unit, University Hospital Basel, Basel, Switzerland; ^4^ Emergency Department, University Hospital Basel, Basel, Switzerland; ^5^ University Center of Cardiovascular Science & Department of Cardiology, University Heart and Vascular Center Hamburg, University Medical Center Hamburg-Eppendorf, Hamburg, Germany; ^6^ Laboratory Medicine, University Hospital Basel, Basel, Switzerland

**Keywords:** COVID-19, complement system, endothelial cells, SARS-CoV-2, ICAM-1, VCAM-1, E-Selectin, galectin-3

## Abstract

**Background:**

Thromboinflammation may influence disease outcome in COVID-19. We aimed to evaluate complement and endothelial cell activation in patients with confirmed COVID-19 compared to controls with clinically suspected but excluded SARS-CoV-2 infection.

**Methods:**

In a prospective, observational, single-center study, patients presenting with clinically suspected COVID-19 were recruited in the emergency department. Blood samples on presentation were obtained for analysis of C5a, sC5b-9, E-selectin, Galectin-3, ICAM-1 and VCAM-1.

**Results:**

153 cases and 166 controls (suffering mainly from non-SARS-CoV-2 respiratory viral infections, non-infectious inflammatory conditions and bacterial pneumonia) were included. Hospital admission occurred in 62% and 45% of cases and controls, respectively. C5a and VCAM-1 concentrations were significantly elevated and E-selectin concentrations decreased in COVID-19 out- and inpatients compared to the respective controls. However, relative differences in outpatients vs. inpatients in most biomarkers were comparable between cases and controls. Elevated concentrations of C5a, Galectin-3, ICAM-1 and VCAM-1 on presentation were associated with the composite outcome of ICU- admission or 30-day mortality in COVID-19 and controls, yet more pronounced in COVID-19. C5a and sC5b-9 concentrations were significantly higher in COVID-19 males vs. females, which was not observed in the control group.

**Conclusions:**

Our data indicate an activation of the complement cascade and endothelium in COVID-19 beyond a nonspecific inflammatory trigger as observed in controls (i.e., “over”-activation).

## Introduction

Coronavirus disease 2019 (COVID-19) includes a variety of clinical pictures ranging from oligosymptomatic upper airway disease to severe disease presenting with a hyperinflammatory state and leading to acute respiratory distress syndrome in up to 31% of the patients (1). Thromboinflammation is a hallmark of COVID-19 and has a major impact on COVID-19 morbidity and mortality (2). Both, activation of the complement system and endothelial cell dysfunction after SARS-CoV-2 infection have been identified as important contributors (3).

The complement system is a central part of the innate immune response. Its activation has been demonstrated upon viral infections and its importance in the immune defense is underscored by strategies of viruses to disable and exploit complemental activation (4). SARS-CoV-2 was shown to directly activate both, the alternative and the lectin pathway of the complement system (5–7), and also the classical pathway *via* SARS-CoV-2 antibodies (8). Besides its beneficial effects, overactivation of complement may result in host damage. Improved outcome and reduced pulmonary tissue damage was demonstrated in murine models upon coronavirus infection, when complement system activation was blocked (9). Histopathology findings revealed extensive deposits of complement components C5b-9, C4d, and mannose-binding lectin serine protease 2 (MASP-2) in COVID-19 patients who died of respiratory failure (10). A subset of clinical studies reported elevated complement activation products in sera of COVID-19 patients that positively correlated with disease severity and were associated with adverse outcomes (11), but comparative data in disease controls are scarce.

Various pathways are involved in endothelial cell activation in COVID-19. Complement components C5a and C5b-9 were shown to induce downstream thromboinflammatory responses, resulting in endothelial activation and damage (12, 13). SARS-CoV-2 tropism for endothelial cells is suggested to further contribute to endothelial activation and dysfunction (14, 15) causing thromboembolism, tissue ischemia and impaired barrier function (10, 16). Upon infection and activation, endothelial surface adhesion molecules E-selectin, intercellular cell adhesion molecule-1 (ICAM-1) and vascular cell adhesion molecule-1 (VCAM-1) are upregulated. E-selectin mediates rolling of leucocytes while ICAM-1 and VCAM-1 are further involved in firm leucocyte adhesion (17, 18) initiating their transendothelial migration to the site of inflammation. Soluble forms of ICAM-1, VCAM-1 and E-selectin are used as biomarkers for endothelial cell activation, and several studies demonstrate increased concentrations of these proteins in COVID-19 patients related to the disease severity (19–21).

A prothrombotic state is the result of an inflammatory feedback loop secondary to complement activation, endothelial cell injury and hypercoagulability (4). Therefore, the interaction between complement and endothelial cell activation is of interest, considering possible therapeutic options in COVID-19 (16, 22, 23). Male sex is a relevant risk factor for mortality in COVID-19 (24) and higher levels of chemokines and cytokines that firmly interact with the complement system (25, 26) were found in male COVID-19 patients vs females (27). Thus, therapeutic options may differ between males and females, respectively.

Another regulator of the inflammatory-associated immune response is Galectin-3, a member of the beta-galactoside binding-protein family with pleiotropic effects, including cell-proliferation, apoptotic regulation and fibrosis (28). Preliminary evidence implies a role for galectin-3 as biomarker and target in COVID-19 due to its upregulation in severe COVID-19 (29, 30) and potential mediator for viral adhesion (31).

Although elevated markers of complement and endothelial activation as well as elevated Galectin-3 concentrations were demonstrated in COVID-19 patients, most studies compared cases with healthy controls, and therefore may overestimate the specificity of the innate immune response upon SARS-CoV-2 infection.

In this prospective cohort study, we primarily evaluated specificity and possible interactions of complement activation and markers of endothelial cell activation in COVID-19 patients by comparing cases to a control population with symptoms suggestive of COVID-19 but tested negative for SARS-CoV-2 infection. Secondarily we analyzed associations of markers with adverse outcomes and investigated patterns of marker activation as well as sex differences in cases and controls.

## Materials and methods

### Patient inclusion, sample collection and data extraction

The COVIVA study was a prospective, observational cohort study that recruited unselected patients aged 18 years and older presenting with symptoms consistent with COVID-19 to the emergency department (ED) of the University Hospital Basel, Switzerland, from March 2020 to June 2020. Patients were considered SARS-CoV-2 infected if at least one SARS-CoV-2 PCR from a nasopharyngeal swab performed at ED presentation or within 14 days prior to or after was positive in combination with consistent clinical signs and symptoms. Patients that were tested negative for SARS-CoV-2 infection by PCR were considered as controls. Adjudication of the final (non-COVID-19) diagnosis was performed by five board-certified physicians that reviewed all medical data available including 30 days post-discharge follow-up information.

All patients underwent a clinical assessment by the treating ED physician according to local standard operating procedures. We obtained blood samples within 24 hours after presentation to the ED. Samples were transferred to the laboratory immediately after sampling, centrifuged, aliquoted and stored at −80°C until measurement.

Patients were contacted by telephone or in written form 30 days after discharge for follow-up. Records of hospitals and primary care physicians as well as national death registries were screened, if applicable.

For the present analysis, blood samples with sufficient residual volume to determine the investigational biomarkers were available from 153/191 COVID-19 patients, which served as cases. Subsequently samples from 166/890 SARS-CoV-2 negative patients were randomly selected to serve as controls. Overall, the numbers of available samples for C5a, sC5b-9, E-selectin, Galectin-3, ICAM-1 and VCAM-1 were 302, 302, 290, 289, 290 and 290, respectively.

### Laboratory assessment

Concentrations of ICAM-1, VCAM-1, Galectin-3 and E-selectin in serum samples were determined by a multiplexed enzyme-linked immunosorbent assay according to the manufacturer’s instruction (proteinsimple, Bio-techne, Minneapolis, MN, U.S.A.). C5a and sC5b-9 concentrations were quantified using enzyme-linked immunosorbent assay (ELISA) kits according to the manufacturer’s instruction (sC5b-9: BD Biosciences Pharmingen, San Diego, CA, U.S.A.; C5a: ThermoFisher Scientific, Waltham, MA, U.S.A.).

### Primary and secondary endpoints

Primary endpoint of this study was the comparison of the systemic concentration of complement activation products (C5a, C5b-9), endothelial activation markers (ICAM-1, VCAM-1, E-selectin) and Galectin-3 in COVID-19 patients and in patients with suspected but excluded SARS-CoV-2 infection, in the out- and inpatient setting.

Secondary endpoints included the association of markers with the composite endpoint of admission to the intensive care unit (ICU) or death within a 30-day follow-up period and with thromboembolic events. Correlations with other laboratory inflammatory markers and sex differences were also analyzed.

### Statistical analysis

Statistical analyses were performed using SPSS software, version 25 (IBM, USA), and data were visualized with GraphPad Prism 7 software (GraphPadSoftwares Inc., La Jolla, Ca, USA). Data are expressed as medians and interquartile range (IQR) for continuous variables, and as numbers and percentages (%) for categorical variables. Continuous variables showed mostly skewed distributions and were compared by Mann-Whitney U test, and Spearman’s rank test was used to evaluate correlations. For nominal variables Chi-squared test and Fisher’s exact test was used.

### Ethics statement

All participating patients or their legally authorized representatives consented by signing a local general consent form. This study was conducted according to the principles of the Declaration of Helsinki and approved by the Ethics Committee of Northwestern and Central Switzerland (EKNZ 2020-00566).

## Results

### Baseline characteristics in COVID-19 cases and controls

Overall, 1086 patients were recruited presenting with symptoms suspicious for COVID-19 from March 2020 to June 2020, and 153/191 COVID-19 and 166/890 randomly selected controls were included in the present analysis. Hospital admission was required in 95 (62%) and 75 (45%) of COVID-19 patients and controls, respectively. Admission to the ICU or death at day 30 occurred in 41 (43%) inpatient cases and 27 (36%) inpatient controls. COVID-19 inpatients were younger than inpatient controls (median age 64 [52, 75] vs. 71 [59, 80] years, p=0.02) and stayed longer in the hospital (8 [5.0, 15.0] vs. 5 [2.0, 11.0] days, p=0.01). Controls suffered mainly from viral infections other than COVID-19 (n = 67, 40%), non-infectious inflammatory conditions (n=40, 25%) and bacterial pneumonia (n = 24, 14%). The demographic and clinical characteristics of the two groups are shown in **Table 1**.

**Table 1:** Baseline characteristics.

	Outpatients			Inpatients		
Variables	COVID-19 (n = 58)	Controls (n = 91)	P value	COVID-19 (n = 95)	Controls (n = 75)	P value
	*Demographics*					
**Age, years**	47 (35 - 59)	46 (32 - 59)	0.661	64 (52 - 75)	71 (59 - 80)	**0.020**
**Male gender**	31 (53)	53 (58)	0.565	61 (64)	42 (56)	0.277
	*Comorbidities*					
**Cardiac disease**	5 (9)	12 (13)	0.393	29 (31)	37 (49)	**0.012**
**Hypertension**	13 (22)	30 (33)	0.166	54 (57)	45 (60)	0.678
**Pulmonary disease**	11 (19)	29 (32)	0.083	19 (20)	22 (29)	0.158
**- Asthma**	10 (17)	20 (22)	0.482	8 (8)	5 (7)	0.669
**- COPD**	0 (0)	6 (7)	0.082	8 (8)	11 (15)	0.199
**Active or previous smoker**	16 (28)	36 (40)	0.135	36 (38)	31 (41)	0.649
**Chronic kidney disease**	0 (0)	2 (2)	0.521	24 (25)	28 (37)	0.090
**Diabetes mellitus**	3 (5)	7 (8)	0.741	28 (30)	17 (23)	0.318
**Immunodeficiency**	2 (3)	6 (7)	0.484	6 (6)	7 (9)	0.462
**Cancer**	4 (7)	1 (1)	0.076	10 (11)	10 (13)	0.573
	*Symptoms and scores at ED*					
***Cough***	41 (71)	63 (69)	0.850	60 (63)	36 (48)	**0.048**
**Dyspnea**	23 (40)	53 (58)	**0.027**	40 (42)	41 (55)	0.103
**Blood oxygen saturation, %**	98 (97-99)	98 (97 - 99)	0.378	95 (93 - 97)	96 (95 - 98)	**0.007**
**NEWS score**	1 (0 - 3)	1 (0 - 2)	0.079	4 (2 - 6)	3 (3 - 6)	0.347
**Time from first symptom to ED presentation, days**	7 (3 - 14)	5 (2 - 10)	**0.021**	7 (3 - 11)	6 (1-6)	**<0.001**
	*Laboratory findings*					
**CRP, mg/L**	2.1 (0.9 – 11.0)	1.2 (0.5 - 6.8)	**0.049**	70.1 (32.8 – 145.7)	49.8 (8.2 – 114.9)	0.053
**D-Dimer, mg/L**	0.38 (0.29 - 0.65)	0.29 (0.29 - 0.39)	**<0.001**	0.87 (0.51 - 2.00)	1.21 (0.63 - 3.46)	0.134
**Ferritin, µg/L**	205 (99 – 395)	137 (76 – 248)	**0.020**	720 (374 – 1238)	273 (114 – 667)	**<0.001**
**Leukocytes, G/L**	5.8 (4.5 - 7.0)	8.1 (6.3 - 10)	**<0.001**	6.7 (5.1 - 8.9)	11.1 (8.3 - 14.8)	**<0.001**
**Lymphocytes, G/L**	1.37 (0.96 - 1.97)	1.80 (1.45 - 2.54)	**<0.001**	0.95 (0.61 - 1.26)	1.02 (0.76 - 1.42)	0.084
**LDH, U/L**	206 (178 – 236)	199 (179 – 227)	0.453	325 (257 – 447)	236 (190 – 362)	**<0.001**
**eGFR, mL/min/1.73m^2^**	99 (89 – 111)	99 (83 – 113)	0.962	78 (49 – 97)	69 (41 – 96)	0.591
	*Patient management and outcomes*					
**Length of hospital stay, days**	0	0		8.0 (5.0 – 15.0)	5.0 (2.0 – 11.0)	**0.010**
**ICU admission or death within 30 days**	0	0		41 (43)	27 (36)	0.327
**Intubation**	0	0		29 (31)	18 (24)	0.345
**Intubation, days**	0	0		9.0 (5.5 – 12.5)	3.0 (1.0 – 7.3)	0.088

Data are expressed in numbers (percentage) or median (interquartile range). Values are shown in bold in case of significant difference.

COPD, chronic obstructive pulmonary disease; NEWS score, National Early Warning Score; ED, emergency department; CRP, C-reactive protein; LDH, lactate dehydrogenase; eGFR, estimated glomerular filtration rate; ICU, intensive care unit.

### Serum levels of Galectin-3, complement and endothelial cell activation markers in COVID-19 cases and controls

Overall, C5a and VCAM-1 concentrations were elevated and E-selectin concentrations decreased in COVID-19 cases compared to controls (p<0.001 for all markers, **Table 2**) with a borderline difference in sC5b-9 levels (p=0.037). These differences were also evident when outpatients and inpatients were separately analyzed with the exception of sC5b-9 in the outpatient group (**Table 2**). Similarly, control patients with a diagnosis of bacterial pneumonia or other respiratory viral infections showed lower C5a and VCAM-1 concentrations compared to COVID-19 cases despite a slightly higher CRP concentration, whereas an increase in E-selectin concentrations was mainly observed in patients with bacterial pneumonia and was less pronounced in patients with other respiratory viral infections (**Supplementary Tables 1, 2**). No significant differences in ICAM-1 and Galectin-3 levels were observed in any analyses.

**Table 2:** Concentrations of markers of complement and endothelial cell activation on admission.

Variable	Overall			Outpatients			Inpatients		
	COVID-19 (n=153)	Controls (n=166)	P value	COVID-19 (n=58)	Controls (n=91)	P value	COVID-19 (n=95)	Controls (n=75)	P value
C5a, ng/ml	3.23 (1.46 – 5.95)	0.91 (0.41 – 1.93)	**<0.001**	1.69 (0.93 - 3.61)	0.57 (0.11 – 1.28)	**<0.001**	4.20 (2.31 – 7.05)	1.46 (0.78 – 2.75)	**<0.001**
sC5b-9, ng/ml	1139 (720 – 1679)	992 (744 – 1315)	**0.037**	1001 (642 - 1364)	954 (719 – 1277)	0.922	1268 (783 – 1905)	1019 (792 – 1391)	**0.026**
E-selectin, ng/ml	25.1 (17.6 – 34.6)	30.5 (22.4 -42.4)	**<0.001**	22.0 (14.5 – 32.1)	28.1 (19.8 -39.9)	**0.005**	26.6 (19.2 – 37.9)	36.8 (25.3 – 51.1)	**0.001**
Galectin-3, ng/ml	9.3 (7.1 – 12.3)	8.5 (6.9 – 10.8)	0.100	7.3 (6.0 – 8.2)	7.5 (6.4 – 9.5)	0.239	11.5 (8.4 – 14.2)	10.6 (8.2 – 12.6)	0.174
ICAM-1, ng/ml	386 (319 -497)	374 (292 – 500)	0.340	330 (279 – 403)	321 (281 – 421)	0.866	442 (353 – 544)	456 (366 – 590)	0.387
VCAM-1, ng/ml	961 (675 – 1434)	625 (493 – 853)	**<0.001**	677 (556 – 862)	532 (445 – 654)	**<0.001**	1276 (908 – 1566)	840 (662 – 1229)	**<0.001**

Median (interquartile range), values are shown in bold in case of significant difference.

Almost all markers were significantly elevated in inpatients vs. outpatients in both groups, and relative increases in inpatients vs. outpatients were comparable in cases and controls (**Table 3**).

**Table 3:** Relative differences in inpatients vs. outpatients.

Variable	Relative difference COVID-19*	P value	Relative difference Controls*	P value	Δ relative differences cases vs. controls in %
C5a	↑ 2.5	<0.001	↑ 2.6	**<0.001**	-3
C5b-9	↑ 1.3	0.003	↔ 1.1	0.343	19
E-Selectin	↑ 1.2	0.016	↑ 1.3	**0.003**	-8
Galectin-3	↑ 1,6	<0.001	↑ 1.4	**<0.001**	12
ICAM-1	↑ 1.3	<0.001	↑ 1.4	**<0.001**	-6
VCAM-1	↑ 1.9	<0.001	↑ 1.6	**<0.001**	19

*Arrows indicate direction, numbers indicate factor of elevation in in- vs. outpatients. Values are shown in bold in case of significant difference.

### Association of Galectin-3, complement and endothelial cell activation markers with outcomes

Overall, elevated concentrations of C5a, Galectin-3, ICAM-1 and VCAM-1 levels on presentation were associated with the composite outcome of ICU admission or 30-day mortality in cases and controls (**Table 4**), as were concentrations of D-Dimer and the inflammatory markers C-reactive protein (CRP) and ferritin. However, when focusing only on inpatients (the composite outcome did not occur in outpatients), elevated concentrations of these activation markers were only predictive of the composite outcome in cases, but not in controls (**Supplementary Table 3**).

**Table 4:** Concentrations of markers of complement and endothelial cell activation and inflammation on admission according to the composite outcome of admission to the intensive care unit or death at 30 days in cases and controls.

	COVID-19, composite outcome			Controls, composite outcome		
Variables	Yes (n=41)	No (n=112)	P value	Yes (n=27)	No (n=139)	P value
C5a, ng/ml	4.46 (3.31 – 7.61)	2.52 (1.38 – 5.55)	**0.001**	1.46 (0.80 – 2.16)	0.82 (0.27 – 1.76)	**0.024**
sC5b-9, ng/ml	1244 (767 – 1929)	1110 (709 – 1533)	0.119	955 (783 – 1131)	1000 (733 – 1333)	0.79
E-selectin, ng/ml	30.9 (19.7 – 47.9)	24.0 (17.4 – 34.1)	0.059	36.8 (25.5 – 59.2)	29.8 (21.9 – 41.6)	0.064
Galectin-3, ng/ml	12.1 (10.3 – 15.9)	8.1 (6.5 – 11.3)	**<0.001**	10.9 (8.3 – 14.2)	8.0 (6.7 – 10.5)	**0.005**
ICAM-1, ng/ml	486 (377 – 662)	366 (301 – 472)	**<0.001**	452 (380 – 614)	358 (289 – 478)	**0.003**
VCAM-1, ng/ml	1323 (1029 – 1699)	835 (654 – 1329)	**<0.001**	863 (678 – 1340)	591 (483 – 797)	**0.001**
CRP, mg/L	112 (48 – 163)	16.9 (1.5 – 52.3)	**<0.001**	57.3 (30.4 – 127.4)	3.2 (0.6 – 33.0)	**<0.001**
D-Dimer, mg/L	1.16 (0.53 – 2.84)	0.52 (0.34 – 1.08)	**0.002**	1.41 (0.56 – 4.89)	0.37 (0.29 – 0.89)	**<0.001**
Ferritin, µg/L	1200 (445 – 2055)	351 (151 – 633)	**<0.001**	256 (115 – 865)	161 (80 – 310)	**0.039**

Median (interquartile range), values are shown in bold in case of significant difference.

CRP, C-reactive protein.

In patients with the composite outcome, concentrations of most markers were significantly higher in COVID-19 cases compared to controls with the exception of D-Dimer levels. In particular, median C5a and ferritin concentrations were markedly elevated (C5a, 4.46 ng/ml in COVID-19 cases vs 1.46 ng/mL in controls, p<0.001; ferritin, 1200 µg/L vs. 256 µg/L, p<0.001).

The analysis of thromboembolic events was limited by the low number of events (n=13). Increased median Galectin-3 and ICAM-1 levels were associated with thromboembolic events in both, cases and controls (**Supplementary Table 4**), whereas associations of increased median C5a and E-selectin concentrations with thromboembolic events were only observed in controls.

### Correlations of Galectin-3, markers of complement and endothelial cell activation and inflammation

**Figure 1:**
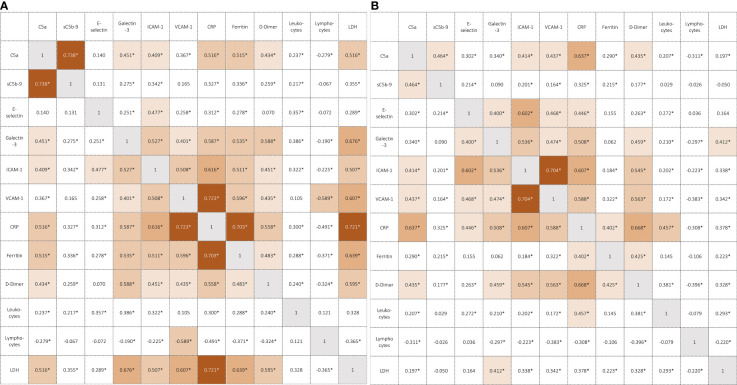
**(A)** Correlation coefficients in COVID-19 patients. *p<0.01 (Spearman correlation coefficient), CRP, C-reactive protein; LDH, lactate dehydrogenase. **(B)** Correlation coefficients in controls. *p<0.01 (Spearman correlation coefficient), CRP, C-reactive protein; LDH, lactate dehydrogenase.

C5a levels correlated moderately with markers of endothelial cell activation and Galectin-3 in both, COVID-19 cases and controls, whereas the correlation was weaker for sC5b-9 (**Figures 1A, B**). C5a, ICAM-1, VCAM-1 and Galectin-3 correlated moderately to strongly with CRP and D-Dimer concentrations in cases and controls, whereas correlation with ferritin and LDH was much weaker in controls. E-selectin correlated strongly with ICAM-1 and VCAM-1 in controls (r = 0.602 and r = 0.468, p<0.01), while a weaker correlation was found in cases (r = 0.477 and r = 0.258, p<0.01).

### Higher levels of complement and inflammatory markers in males vs. females (inpatients)

Both, median C5a and sC5b-9 concentrations were higher in COVID-19 males vs. females (C5a, 4.62 vs. 3.46 ng/ml, p=0.019; sCb5-9, 1476 ng/ml vs 859 ng/mL, p=0.008) which was not observed in the control group. Ferritin was significantly higher in males vs. females in both, COVID 19 cases and controls, whereas significantly higher CRP levels in males vs. females were only observed in COVID-19 cases (**Table 5**). Endothelial cell activation markers were similarly distributed with the exception of higher VCAM-1 levels in COVID-19 males vs. females (data not shown).

**Table 5:** Sex differences of complement activation and inflammatory markers in COVID-19 inpatients vs. controls on admission.

	COVID-19			Controls		
Variables	Male (n = 61)	Female (n=34)	P value	Male (n=42)	Female (n=33)	P value
C5a, ng/ml	4.62 (2.67 – 9.15)	3.46 (1.47 – 5.59)	**0.019**	1.91 (0.99 – 4.02)	1.27 (0.43 – 2.06)	0.054
sC5b-9, ng/ml	1476 (816 – 2023)	859 (718 – 1486)	**0.008**	1044 (802 – 1384)	986 (591 – 1449)	0.852
CRP, mg/L	86.7 (35.3 – 169.4)	56.9 (25.3 – 81.5)	**0.025**	56.4 (28.6 – 151)	39.7 (3.7 – 92.2)	0.180
Ferritin, µg/L	930 (511 – 1397)	392 (199 -728)	**<0.001**	463 (174 – 856)	148 (67 – 468)	**0.004**

Median (interquartile range), values are shown in bold in case of significant differences.

CRP, C-reactive protein.

## Discussion

Thromboinflammation has been shown to contribute substantially to the pathogenesis and disease severity in COVID-19 (2). The present study is the largest study to date to assess complement activation markers C5a, C5b-9 as well as endothelial cell activation markers E-selectin, ICAM-1 and VCAM-1 and Galectin-3, an immunoregulatory protein, in COVID-19 cases and SARS-CoV-2 negative controls with similar symptoms and presentation. With few exceptions (32, 33), most previous studies had chosen healthy individuals as controls, thereby potentially overestimating the specificity of those biomarkers for COVID-19.

Indeed, while concentrations of C5a and VCAM-1 were higher and E-selectin concentrations lower in COVID-19 cases vs. the respective controls, the relative difference when comparing inpatients vs. outpatients was comparable in cases and controls. Moreover, these markers correlated equally well with other inflammatory proteins such as CRP or D-Dimer. This is consistent with an unspecific increase of complement and endothelial cell activation depending on severity. As such the increase in complement and endothelial cell activation may be regarded as nonspecific for SARS-CoV-2 and rather reflect the clinical picture it causes.

However, despite a similar degree of inflammation (as assessed by CRP and D-Dimer levels), C5a and VCAM-1 concentrations were significantly higher and E-selectin concentrations significantly lower in COVID-19 cases compared to control patients with bacterial pneumonia pointing towards a differential response in COVID-19 patients. Hence, increased complement and endothelial cell activation seems to be a distinctive feature of COVID-19 that cannot be solely explained by a higher inflammatory response. Interestingly, their correlation with ferritin, a protein that has been identified as a promising outcome predictor in severe COVID-19 (34), was stronger in COVID-19 patients compared to controls again arguing for a specific response of the analyzed markers in SARS-CoV-2 infection.

Recently, Ma et al. (33) showed elevated sC5b-9 in severe COVID-19 compared to those hospitalized with influenza or other forms of acute respiratory failure. Here we demonstrated elevated levels of C5a and sC5b-9 in COVID-19 patients compared to a broad control population in an inpatient and outpatient setting, consisting mostly of patients with viral respiratory infection other than COVID-19, bacterial pneumonia but also with systemic inflammatory response syndromes secondary to non-infectious etiologies. Notably, sC5b-9 was markedly elevated in inpatient cases vs controls (1268 vs 1019 ng/mL, p=0.026) while no relevant difference was found in outpatients. These findings may reflect increasing involvement of sC5b-9 in COVID-19 in severe disease. Histopathologic findings support the hypothesis of C5b-9-mediated endotheliopathy in COVID-19, induced by C5b-9 pore formation on endothelial membrane (35). Our findings underscore the growing evidence (36) of the involvement of the cytolytic complement system in severe COVID-19 and the assumption that the membrane attack complex is a major contributor to observed endotheliopathy.

Bauer et al. showed elevated VCAM-1 concentrations, while ICAM-1 and E-selectin did not differ in cases vs. disease controls. In line with these findings, Cugno and Yao (19, 37) showed no difference in E-selectin concentrations between mild to moderate COVID-19 cases and healthy controls. Our results regarding the cellular adhesion molecules are in line with these studies, but we demonstrate markedly lower E-selectin concentrations in cases vs. controls, mostly driven by control patients with bacterial pneumonia. Previous studies reported substantially higher E-selectin concentrations in patients with bacterial sepsis (18). Still, E-selectin concentrations were also higher in outpatient controls with viral respiratory infections compared to COVID-19 cases, and thus a lower degree of E-selectin production may be regarded as specific feature of COVID-19.

Previous studies showed increased Galectin-3 plasma levels in patients with COVID-19 compared to healthy controls (29, 30). In the present study, we did not observe a difference in Galectin-3 concentrations in cases and disease controls. We assume that previous findings of elevated Galectin-3 in patients with COVID-19 compared to healthy controls are not specific for COVID-19 but reflects a nonspecific inflammatory response. However, Galectin-3 binding protein was recently identified as elevated in critically ill COVID-19 patients only (and not in disease controls) and to correlate with complement proteins and regulators (38). Hence, Galectin-3 may be considered as a therapeutic target upon SARS-CoV-2 infection due to its possible role in viral adhesion, and its association with disease severity and adverse outcome as demonstrated in the present study.

Associations of elevated complement and endothelial activation markers with COVID-19 outcome or adverse events were previously described (20, 21, 33, 39–41). At first sight, the association of elevated C5a, ICAM-1 and VCAM-1 concentration on admission with ICU admission or 30-day mortality did not point towards a specific feature of COVID-19, as it was observed in cases and controls in the present study. However, the association was not evident in controls anymore, when focusing on inpatients only. Likewise, the magnitude of C5a elevation was more pronounced in COVID-19 cases progressing towards the composite outcome compared to disease controls. Hence, our data are consistent with an additional activation of the complement system and endothelial cells in COVID-19 beyond a nonspecific baseline inflammatory activation of these systems (i.e., “over”-activation) in COVID-19. This has also been described (and confirmed in the present study) for ferritin levels (42). Although complement activation may serve as predictive marker for adverse outcomes, indicators such as CRP, ferritin or D-Dimer are more convenient, as they are equally associated with adverse outcomes (43) and correlate at least moderately with complement activation.

A relevant risk factor for mortality in COVID-19 is male sex which is not completely explained by a higher prevalence of comorbidities in men (24). Differences in sex chromosome genes (immune response X-linked genes and disease susceptibility genes), sex hormones and expression of ACE2 receptors are discussed as underlying cause of different outcomes in COVID-19 (44). Takahashi et al. found that levels of pro-inflammatory innate immunity chemokines and cytokines, such as IL-8, IL-18 and CCL5 were higher in male patients (27). C5a strongly amplifies IL-8 expression (26) and complement was shown to elicit secretion of IL-18 (25). Mussini et al. suggested that C-reactive protein explains 85% of the different incidence in invasive mechanical ventilation or death between male and females, which was to a lesser extent the case for ferritin and LDH. They proposed a differential activation of the innate immune response, mediated by CRP, to explain the observed sex difference in COVID-19 (45). CRP is known to aggravate the inflammatory response by activating the classical complement pathway culminating in the cleavage of C3 and C5 generating anaphylatoxins such as C5a. We observed higher CRP, C5a and sC5b-9 levels on admission in COVID-19 males compared to females, which was not evident in the control population. These results suggest a different level of activation of the complement system in males vs. females upon SARS-CoV-2 infection. Future studies are required to elucidate if differences in alternative pathway activation in males vs. females, as observed in healthy individuals (46), may drive the observed excessive activation in males and may explain sex difference in clinical outcomes in COVID-19. Of note, an overactivated alternative pathway has recently been implicated in critically ill COVID-19 patients (47).

Our study has several limitations. First, the single-center design limits external validity. Second, COVID-19 cases and controls were not matched, hence some differences were significant suggesting that COVID-19 inpatients may have had less comorbidities. Third, only one sample was drawn on admission for most patients limiting the analysis of the time course of the investigated parameters. As an example, E-selectin concentrations are known to decrease shorty after induction. A major strength of this study is the prospective design with enrolment of consecutive patients presenting to the ED with suspicion of COVID-19 and adjudication of the final diagnosis by five board-certified physicians.

In conclusion, the present study confirms increased complement and endothelial cell activity in COVID-19 patients compared to disease controls. Our data are consistent with an additional activation of the complement cascade and endothelial cells in COVID-19 exceeding a rather nonspecific baseline inflammatory trigger as observed in disease controls (i.e., “over”-activation). The observed increase in complement activation markers in male vs. female COVID-19 patients requires further studies.

## Data availability statement

The raw data supporting the conclusions of this article will be made available by the authors, without undue reservation.

## Ethics statement

The studies involving human participants were reviewed and approved by the Ethics Committee of Northwestern and Central Switzerland. The patients/participants provided their written informed consent to participate in this study.

## Author contributions

FB and MO had full access to all of the data in the study and take responsibility for the integrity of the data and the accuracy of the data analysis. All authors contributed substantially to the study design and interpretation. FB, ML, and RT collected the data. FB and MO analyzed the data. FB and MO prepared a first manuscript draft. All authors contributed substantially to the writing of the manuscript, critically revised the manuscript for important intellectual content and approved the final version.

## Funding

The COVIVA study was supported by the Swiss Heart Foundation, the Cardiovascular Research Foundation Basel, and an unrestricted research grant by Roche Diagnostics. The presented analysis was however funded by departmental funds of the corresponding author.

## Conflict of interest

MO reports research support from the Swiss National Science Foundation (Grant No 32003B_197880) and Pharming Biotechnologies B.V. outside the submitted work. RT reports research support from the Swiss National Science Foundation (Grant No P300PB_167803), the Swiss Heart Foundation, the Swiss Society of Cardiology, the Cardiovascular Research Foundation Basel, the University of Basel and the University Hospital Basel and speaker honoraria/consulting honoraria from Abbott, Amgen, Astra Zeneca, Roche, Siemens, Singulex, and Thermo Scientific BRAHMS.

The remaining authors declare that the research was conducted in the absence of any commercial or financial relationships that could be construed as a potential conflict of interest.

The handling editor (PN) declared a shared committee with the author (MT) at the time of review.

## Publisher’s note

All claims expressed in this article are solely those of the authors and do not necessarily represent those of their affiliated organizations, or those of the publisher, the editors and the reviewers. Any product that may be evaluated in this article, or claim that may be made by its manufacturer, is not guaranteed or endorsed by the publisher.
