# Supplementary material for: Complement and endothelial cell activation in COVID-19 patients compared to controls with suspected SARS-CoV-2 infection: A prospective cohort study
Source: Front Immunol. 2022 Sep 20;13:941742. doi: 10.3389/fimmu.2022.941742 (PMC9530900; doi:10.3389/fimmu.2022.941742)
Supplement: Supplementary file 1 [file Table_1.docx]

Supplementary Content

Table S1 Concentrations of markers of complement and endothelial cell activation on admission in inpatients with COVID-19, bacterial pneumonia, and respiratory viral infection

| Variables | COVID-19 n=95 | Bacterial Pneumonia n=22 | p-value | Respiratory Viral Infection n=13 | p-value |
| --- | --- | --- | --- | --- | --- |
| C5a, ng/ml | 4.20 (2.31 – 7.05) | 2.19 (1.26 – 3.52) | **<0.001** | 1.49 (0.87 – 1.97) | **< 0.001** |
| sC5b-9, ng/ml | 1268 (783 – 1905) | 1105 (759 – 1487) | 0.248 | 966 (742 – 1122) | 0.25 |
| E-selectin, ng/ml | 26.6 (19.2 – 37.9) | 44.3 (27.0) | **0.001** | 28.3 (22.3 – 51.1) | 0.118 |
| Galectin-3, ng/ml | 11.5 (8.4 – 14.2) | 10.7 (9.4 -13.0) | 0.575 | 10.8 (8.5 – 12.6) | 0.428 |
| ICAM-1, ng/ml | 442 (353 – 544) | 500 (375 – 583) | 0.245 | 436 (363 – 489) | 0.745 |
| VCAM-1, ng/ml | 1276 (908 – 1566) | 835 (715 – 1238) | **0.016** | 653 (499 – 1095) | **0.001** |
| CRP, mg/L | 70.1 (32.8 – 145.7) | 105.6 (39.7 – 175.9) | 0.293 | 34.7 (3.35 – 55.0) | **0.008** |
| D-Dimer, mg/L | 0.87 (0.51 - 2.00) | 1.59 (0.61 – 4.89) | 0.112 | 0.76 (0.56 – 55.0) | 0.852 |
| Ferritin, µg/L | 720 (374 – 1238) | 302.0 (108.5 – 582.0) | **0.005** | 153 (56 – 276) | **<0.001** |

Median (interquartile range), values are shown in bold in case of significant difference

Abbreviations: CRP, C-reactive protein

Table S2 Concentrations of markers of complement and endothelial cell activation on admission in outpatients with COVID-19 and other respiratory viral infection

| Variables | COVID-19  n=58 | Respiratory Viral Infection  n = 54 | p-value |
| --- | --- | --- | --- |
| C5a, ng/ml | 1.69 (0.93 – 3.61) | 0.57 (0.11 – 1.31) | **<0.001** |
| sC5b-9, ng/ml | 1001 (642 – 1364) | 982 (694 -1239) | 0.917 |
| E-selectin, ng/ml | 22.0 (14.5 – 32.1) | 28.2 (19.4 – 41.7) | **0.015** |
| Galectin-3, ng/ml | 7.3 (6.0 – 8.2) | 7.5 (6.1 – 9.7) | 0.312 |
| ICAM-1, ng/ml | 330 (279 – 403) | 322 (281 – 412) | 0.832 |
| VCAM-1, ng/ml | 677 (556 – 862) | 553 (460 -642) | **<0.001** |
| CRP, mg/L | 2.1 (0.9 – 11.0) | 1.35 (0.48 – 7.65) | 0.250 |
| D-Dimer, mg/L | 0.38 (0.29 - 0.65) | 0.29 (0.29 – 0.39) | **<0.001** |
| Ferritin, µg/L | 205 (99 – 395) | 132 (66.75 – 229) | **0.015** |

Median (interquartile range), values are shown in bold in case of significant difference

Abbreviations: CRP, C-reactive protein

Table S3 Concentrations of complement and endothelial cell activation markers according to the composite outcome of admission to intensive care or death at 30 days in inpatient cases and controls.

|  | Inpatient COVID-19 cases, composite outcome | | | Inpatient Controls, composite outcome | | |
| --- | --- | --- | --- | --- | --- | --- |
| Variables | Yes (n=41) | No (n=54) | P value | Yes (n=27) | No (n=46) | P value |
| C5a, ng/ml | 4.46 (3.31 – 7.61) | 3.56 (2.07 – 7.05) | 0.136 | 1.46 (0.80 – 2.16) | 1.45 (0.63 – 3.17) | 0.834 |
| sC5b-9, ng/ml | 1244 (767 – 1929) | 1294 (798 – 1814) | 0.775 | 955 (783 – 1131) | 1084 (780 – 1422) | 0.432 |
| E-selectin, ng/ml | 30.9 (19.7 – 47.9) | 25.8 (19.2 – 34.9) | 0.320 | 36.8 (25.5 – 59.2) | 40.0 (25.0 – 47.0) | 0.715 |
| Galectin-3, ng/ml | 12.1 (10.3 – 15.9) | 10.4 (7.8 – 12.9) | **0.014** | 10.9 (8.3 – 14.2) | 10.2 (8.2 – 11.9) | 0.580 |
| ICAM-1, ng/ml | 486 (377 – 662) | 432 (347 – 529) | **0.047** | 452 (380 – 614) | 462 (351 – 570) | 0.570 |
| VCAM-1, ng/ml | 1323 (1029 – 1699) | 1225 (773 – 1517) | 0.075 | 863 (678 – 1340) | 808 (640 – 1112) | 0.466 |
| CRP, mg/L | 112.4 (48.0 – 162.5) | 54.8 (30.6 – 94.2) | **0.012** | 57.3 (30.4 – 127.4) | 43.3 (3.2 – 100.3) | 0.159 |
| D-Dimer, mg/L | 1.16 (0.53 – 2.84) | 0.78 (0.48 – 1.75) | 0.389 | 1.41 (0.56 – 4.89) | 1.16 (0.64 – 2.08) | 0.344 |
| Ferritin, µg/L | 1200 (445 – 2055) | 606 (333 – 934) | **0.004** | 256 (115 – 865) | 273 (114 – 533) | 0.686 |

Median (interquartile range), values are shown in bold in case of significant difference

Abbreviations: CRP, C-reactive protei

Table S4 Concentrations of complement and endothelial cell activation markers according to the occurrence of thromboembolic events in cases and controls.

|  | COVID-19, composite outcome | | | Controls, composite outcome | | |
| --- | --- | --- | --- | --- | --- | --- |
| Variables | Yes (n=8) | No (n=145) | P value | Yes (n=5) | No (n=161) | P value |
| C5a, ng/ml | 3.86 (2.31 – 5.16) | 3.12 (1.41 – 6.22) | 0.464 | 2.29 (1.73 – 5.07) | 0.86 (0.39 – 1.83) | **0.013** |
| sC5b-9, ng/ml | 1188 (832 – 2257) | 1135 (718 – 1679) | 0.724 | 932 (847 – 2513) | 995 (733 – 1307) | 0.441 |
| E-selectin, ng/ml | 24.5 (22.1 – 46.8) | 25.1 (17.5 – 34.3) | 0.470 | 50.9 (36.4 – 74.1) | 30.4 (22.1 – 41.9) | **0.028** |
| Galectin-3, ng/ml | 14.4 (9.2 – 17.2) | 8.9 (6.9 – 11.9) | **0.013** | 11.3 (9.7 – 13.1) | 8.2 (6.7 – 10.7) | **0.031** |
| ICAM-1, ng/ml | 546 (388 – 794) | 381 (314 -481) | **0.010** | 811 (325 – 1338) | 373 (292 – 495) | 0.098 |
| VCAM-1, ng/ml | 1397 (800 – 1873) | 917 (674 – 1398) | 0.105 | 1269 (851 – 2259) | 611 (487 – 838) | **0.006** |
| CRP, mg/L | 79.7 (33.6 – 193.5) | 29.6 (2.5 – 76.3) | **0.035** | 90.6 (14.7-103)* | 4.75 (0.80 – 43.4) | 0.098 |
| D-Dimer, mg/L | 3.27 (0.62 – 5.22) | 0.59 (0.35 – 1.22) | **0.027** | 4.01 (3.91-6.35)* | 0.41 (0.29 – 1.16) | **0.008** |
| Ferritin, µg/L | 1135 (569 – 3330) | 455 (184 – 833) | **0.007** | 619 (192-1892)* | 164 (81 – 352) | 0.064 |

Median (interquartile range) except * (range), values are shown in bold in case of significant difference

Abbreviations: CRP, C-reactive protein
